# Changes in Oxidised Phospholipids in Response to Oxidative Stress in *Microtubule-Associated Protein Tau* (*MAPT*) Mutant Dopamine Neurons

**DOI:** 10.3390/antiox13050508

**Published:** 2024-04-24

**Authors:** Xanthe Bradford, Hugo J. R. Fernandes, Stuart G. Snowden

**Affiliations:** 1 Department of Biological Sciences, Royal Holloway University of London, London TW20 0EX, UK; xanthe.bradford.2020@live.rhul.ac.uk; 2 Department of Clinical Neurosciences, UK Dementia Research Institute, University of Cambridge, Cambridge Biomedical Campus, Cambridge CB2 0AH, UK; hugo.ribeirofernandes@dpag.ox.ac.uk; 3 Kavli Institute for Nanoscience Discovery, University of Oxford, Dorothy Crowfoot Hodgkin Building, South Parks Road, Oxford OX1 3QU, UK; 4 Department of Physiology, Anatomy and Genetics, University of Oxford, Oxford OX1 3QX, UK; 5 Core Metabolomics and Lipidomics Laboratory, Institute of Metabolic Science, University of Cambridge, Level 4 Pathology, Cambridge Biomedical Campus, Cambridge CB2 0QQ, UK

**Keywords:** *microtubule-associated protein tau*, *MAPT*, oxidised phospholipids, oxidative stress, XBP1, unfolded protein response

## Abstract

*Microtubule-associated protein Tau* (*MAPT*) is strongly associated with the development of neurodegenerative diseases. In addition to driving the formation of neurofibrillary tangles (NFT), mutations in the *MAPT* gene can also cause oxidative stress through hyperpolarisation of the mitochondria. This study explores the impact that *MAPT* mutation is having on phospholipid metabolism in iPSC-derived dopamine neurons, and to determine if these effects are exacerbated by mitochondrial and endoplasmic reticulum stress. Neurons that possessed a mutated copy of *MAPT* were shown to have significantly higher levels of oxo-phospholipids (Oxo-PL) than wild-type neurons. Oxidation of the hydrophobic fatty acid side chains changes the chemistry of the phospholipid leading to disruption of membrane function and potential cell lysis. In wild-type neurons, both mitochondrial and endoplasmic reticulum stress increased Oxo-PL abundance; however, in *MAPT* mutant neurons mitochondrial stress appeared to have a minimal effect. Endoplasmic reticulum stress, surprisingly, reduced the abundance of Oxo-PL in *MAPT* mutant dopamine neurons, and we postulate that this reduction could be modulated through hyperactivation of the unfolded protein response and X-box binding protein 1. Overall, the results of this study contribute to furthering our understanding of the regulation and impact of oxidative stress in Parkinson’s disease pathology.

## 1. Introduction

A prominent characteristic of many neurodegenerative diseases, such as Parkinson’s and Alzheimer’s disease, is the aggregation of the microtubule-associated protein Tau. The protein, whose name derives from the acronym of ‘tubulin-associated unit’, functions to assemble and strengthen tubulin into microtubules, allowing normal cytoskeletal stability and organelle trafficking [1]. Mutations in the *MAPT* gene, which codes for Tau, can cause hyperphosphorylation of the protein, leading to a reduction in its affinity for tubulin [2], leading to dysfunction of the cytoskeleton. In addition to microtubule dissociation, this results in an increased cytosolic concentration of free Tau, which subsequently aggregates into neurofibrillary tangles (NFTs). Though the mechanism is unclear, these tangles result in neuronal damage, producing the neurodegeneration seen in tauopathy-associated diseases. In Parkinson’s disease (PD), these NFTs can drive neurodegeneration in dopamine neurons primarily in the substantia nigra, consequently affecting a person’s motor function and cognition [3]. Although the mechanisms underlying the loss of dopamine neurons are still unclear, previous studies implicated a role for mitochondrial and endoplasmic reticulum (ER) dysfunction in PD-patient-derived cells [4,5].

In addition to formation of NFTs, mutations in the *MAPT* gene can also cause oxidative stress; a disruption in the normal cellular balance of reactive oxygen species (ROS) with oxidative stress is well-documented to be involved in the pathogenesis of neurodegeneration. The mechanisms involved in elevated ROS production [6] and neurodegenerative aetiology resulting from *MAPT* mutation may include aberrant mitochondrial and ER function [7] (for example, through mitochondrial hyperpolarisation [8]), disruption of protein folding and inflammatory processes [9], among other mechanisms [10]. 

The pathological burden of mutations in *MAPT*, at least in part, is driven by the significant effects of oxidative stress on all components of the cell. Moreover, ROS can modify the conformation of amino acid residues, leading to a loss of enzymatic activation, protein aggregation, and vulnerability to proteolysis [11,12]. Another major effect of oxidative stress is the oxidation of lipids, with an excess of ROS such as hydroxyl radicals and peroxynitrate actively inducing lipid peroxidation [13]. This triggers a chain reaction of damage to lipids, lipoproteins, and the phospholipid bilayers with major biological implications. For example, in mitochondria, ROS can oxidise and damage the lipid-rich inner mitochondrial membrane and enzymes and proteins involved in the electron transport chain [14], compromising energy production within the cell. 

Previous metabolomic approaches to study tauopathy and *MAPT* mutations took a general view of metabolism rather than focusing on the impact of ROS and formation of oxidised lipid species [15]. Owing to their involvement in a wide range of important biological processes, including neuroinflammation, disruption of the phospholipid membrane integrity and neuronal signalling, it is crucial to understand their contribution to pathology. So, to better understand the mechanisms underlying tauopathy we need to extend our understanding beyond the results of previous studies and explore the role of oxidized phospholipids. This study aimed to identify metabolic dysregulation in iPSC-derived human dopamine neurons carrying a mutation in the *MAPT* gene which may be driving pathogenesis in PD, with a focus on oxidised phospholipids. Additionally, we determined the impact of mitochondrial and ER stressors on lipid peroxidation in both wild-type and *MAPT* mutant dopamine neurons to gain a deeper understanding of the relationship between the microtubule-associated protein Tau, oxidative stress, and phospholipid metabolism. 

## 2. Materials and Methods

The data described in this study were previously published by Fernandes et al. [15], and a detailed description of the methods including the generation of dopamine neurons and metabolic analysis can be found in this paper. The previous publication focused on the effect of mitochondrial and endoplasmic reticulum stress on global metabolic dysregulation in iPSC-derived dopamine neurons, whilst this study focuses directly on the effect of mutations in the gene encoding of the microtubule-associated protein Tau on the formation of oxidised phospholipids. 

### 2.1. Cell Culture, Dopamine Differentiation, and Drug Treatment

Human iPSCs were cultured with TeSR-E8 medium using Vitronectin-coated plates, and cells were passaged with 0.5 mM EDTA at 70% confluency at a ratio of 1:6. Differentiation into dopamine neurons was performed following a modified version of previous protocols [16,17]. In brief, iPSCs were dissociated into single cells plated at a density of 150,000 cells/cm^2^ on Geltrex-coated plates and grown for 11 days in Knockout Serum Replacement media (KSR) containing Knockout DMEM media, Knockout serum replacement (15%), Non-essential Amino Acids (1:100), 2-Mercaptoethanol (10 μM), and 2 mM L-glutamine. KSR medium was gradually changed to NNB medium containing Neurobasal medium, N2 (0.5X), and B27 (0.5X) and 2 mM L-glutamine from day 6 onwards. Media were changed to NB medium on day 12 containing Neurobasal medium, B27 (1X) and 2 mM L-glutamine. Media were supplemented with LDN-193189 (100 nM) from days 0–10; SB431542 (10 μM) from days 0–4; SAG (100 nM) from days 1–6; Purmorphamine (2 μM) from days 1–6; FGF8a (100 ng/mL) from days 1–6; and CHIR99021 (3 μM) from days 3–12. From Day 12 onwards, other supplements were added: BDNF (20 ng/mL), GDNF (20 ng/mL), Ascorbic Acid (200 μM), TGFβ3 (1 ng/mL), dibutyryl cAMP (500 μM), and DAPT (10 μM). At day 21, cells were dissociated with StemPro Accutase and replated at a density of 300,000 cells/cm^2^ in dishes pre-coated with Geltrex with neurons fed every second day for 2 weeks before analysis. After differentiation, dopamine neurons were treated for 24 h with either 5 μM of Tunicamycin or 1 μM of Rotenone [18] to induce endoplasmic reticulum and mitochondrial stress, respectively.

### 2.2. Sample Preparation and LC-MS Analysis

The methods used in this study have been described previously [15,18]. Briefly, 140 µL of methyl tertiary-butyl ether and 40 µL methanol of methanol were added to disrupt cellular membranes, with 30 µL added prior to spinning at 5000× *g* to achieve phase separation. Extraction blanks were obtained by substituting the cell pellet with 15 µL of HPLC grade water, with 5 µL of all samples taken to produce a pooled quality control. LC-MS analysis was performed on an Agilent infinity HPLC system coupled to and Agilent 6550 ion funnel QToF (Agilent, Santa Clara, CA, USA). An Agilent Poroshell C18 column (2.1 × 150 mm, 2.7 µm) was used to separate organic phase metabolites using mobile-phase A 10 mM ammonium formate in water and mobile-phase B 10 mM ammonium formate in MTBE:MeOH (2:1) using a flow rate of 0.425 mL/min with the column held at 55 °C.

### 2.3. Data Processing, Statistical Analysis, and Metabolite Annotation

For this study, data were re-processed, with raw data processed in R using the CAMERA package, parts per million (PPM) set to 30, signal-to-noise (SN) set to 5, and peak width at 8–90. Subsequently, data clean-up was performed in Excel with metabolite features being defined as having a mean abundance of three times greater in biological samples relative to blanks and present in at least 80% of at least one sample group. All analyses were performed in R version 4.2.1 [19]. The prcomp() function was used to conduct a principal components analysis (PCA) to reduce the dimensionality of the dataset while maintaining the key data structure and visualize the general structure to allow the identification of outliers and multivariate quality control. After quality control, a sparse partial least squares-discriminant analysis (sPLS-DA) was conducted to identify covariance within the data, allowing us to extract the changes in metabolite abundance most strongly associated with the genotype. sPLS-DA is a variant of PLS-DA which allows for selection of the most discriminative features of classification that have been identified in the data. In this study, we used *n* = 6 per group, with this size used calculation were made based on our previous metabolomic analyses of cultured dopamine neurons, and assumed a two-sided type 1 error and a CV of 25%; (in line with previous data) a sample size of 6 gives an 87% possibility of detecting a 50% difference in metabolite abundance at an alpha of 0.05. A univariate analysis of metabolites used a Shapiro–Wilks test to assess data distribution. A Mann–Whitney U test was then performed for the analysis of individual metabolites, as it compares the rank sum of samples from two independent groups, in this case the metabolites’ abundance in either wild-type or *MAPT*-mutated samples. As the Shapiro–Wilks test confirmed that the data were non-normally distributed in every metabolite, the Mann–Whitney U test was chosen over an independent samples t-test. Metabolite features were annotated using the Human Metabolome Database (HMDB) with the *m*/*z* of each significant metabolite feature searched, and from the resulting list of metabolite suggestions, putative annotations were made based on the accuracy of the mass match and expected retention time.

## 3. Results

**Figure 1:**
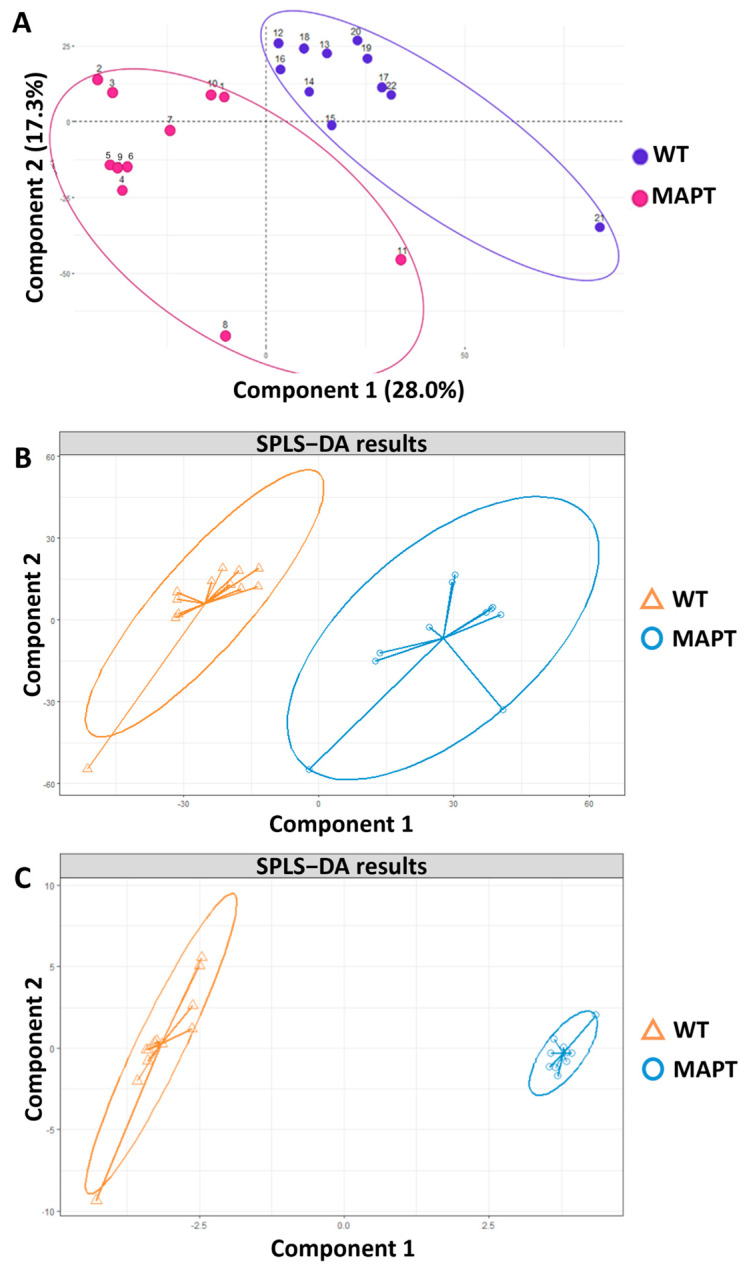
Results of multivariate analysis of metabolites found in human-derived dopamine neuron samples of wild-type or *MAPT* genotype—where each point represents 1 sample. (**A**) PCA scores plot of metabolite abundance data for untreated samples. (**B**) SPLS-DA scores plot for untreated samples. (**C**) SPLS-DA scores plot of untreated samples using only the top 10% of metabolites contributing the most to variance in metabolite data.

In this study, *MAPT*-N279K mutant dopamine neurons (2 lines) were compared to those of isogenic wild-type controls (two lines) to assess the impact of the mutation on the phospholipid metabolism. In addition to this, the impact of mitochondrial and ER stress on these pathways was assessed by treating dopamine neurons with rotenone and tunicamycin, respectively, and comparing them to DMSO-treated controls to identify metabolic shifts. Initial PCA (Supplemental Figure S1) showed that the majority of observed variance (97.8%) on component 1 was attributable to the distance between point 24 (a wild-type sample) and the remaining samples. Component 2 represents the remaining samples accounting for only 0.6% of total variance 163-fold less was explained using this individual sample, making it difficult to interpret patterns among samples. This sample was subsequently removed from the dataset; shown below is the PCA plot following outlier removal (Figure 1A) with samples now showing clustering by genotype.

SPLS-DA supported the findings shown in the PCA, and also revealed clustering by genotype (Figure 1B,C); this was emphasised after variable selection with the top 10% of the explanatory variables (metabolites) kept in the model. This suggests that the metabolites contributing the most to variance in the dataset are the ones that influence even bigger differences by genotype, solidifying the conclusion that the genotype is the biggest factor driving variance in the analysis.

**Figure 2:**
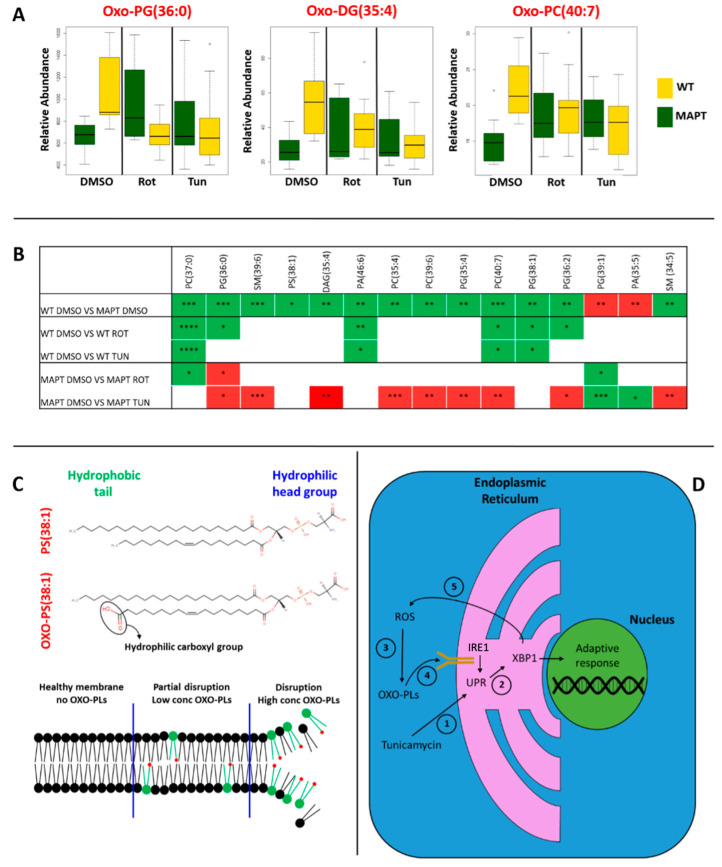
Results showing the effect of the *MAPT*-N279K mutation as well as mitochondrial and endoplasmic reticulum stress on the abundance of oxidized phospholipids (Oxo-PLs) in iPSC-derived dopamine neurons. (**A**) Boxplots showing the abundance of Oxo-PLs. Yellow—wild-type, green—*MAPT* mutated. (**B**) Table showing the effect of *MAPT* mutation, mitochondrial and endoplasmic reticulum stress on individual Oxo-PLs, green = increased in abundance, red = decreased in abundance. (**C**) Schematic showing the impact of Oxo-PLs on membrane integrity. (**D**) Schematic showing proposed modulation of UPR and ROS by tunicamycin: (1) tunicamycin induces ER and oxidative stress. (2) ER stress triggers ER UPR leading to increased XBP1 (a UPR component). (3) High ROS leads to formation of Oxo-PLs. (4) Oxo-PLs activate IRE1 increasing UPR and in turn increasing XBP1. (5) XBP1 acts to reduce ROS and initiates an adaptive response. *p*-values * < 0.05, ** < 0.01, *** < 0.001 **** < 0.0001. Abbreviations: conc—concentration, DAG—Diglyceride, DMSO—dimethylsulfoxide, IRE1—inositol requiring enzyme 1, PA—Phosphatidic acid, PC—Phosphatidylcholine, PG—Phosphatidylglycerol, PS—Phosphatidylglycerol, ROT—Rotenone treated, SM—Sphingomyelin, TUN—Tunicamycin treatment, UPR—unfolded protein response, XBP1—X-box binding protein 1.

In total, we were able to identify and robustly annotate 15 oxidised phospholipids that differed in abundance between cells of differing genotypes, with 13 of these at higher abundances in the *MAPT* mutant neurons with only PG(39:1) and PA(35:5) being more abundant in the wild type (Figure 2B). When we looked at the effect of mitochondrial and endoplasmic reticulum stress in wild-type neurons, we saw that six oxidised phospholipids increased in response to rotenone treatment and four increased in response to tunicamycin. In *MAPT* mutant neurons, mitochondrial stress affected 3 of 15 Oxo-PLs with PC(37:0) and PG(39:1) increasing and PG(36:0) decreasing in abundance (Figure 2B). However, when we look at the impact of ER stress on abundance, 11 of the 15 oxidised phospholipids are affected with only PG(39:1) and PA(35:5) (the two species at lower levels in *MAPT* relative to wild type) increasing in abundance and the remaining nine decreasing (Figure 2B).

## 4. Discussion

Phospholipids are especially vulnerable to oxidative stress [20], with an increase in ROS leading to greater formation of oxidised phospholipids. Thus, the higher levels of Oxo-PLs seen in this study supports an increase in oxidative stress in human dopamine neurons carrying an *MAPT* mutation. With phospholipids representing a major component of all cellular membranes, it is important to understand how oxidation of these components effect’s function. The phospholipids in the bilayer arrange with the polar head group on the exterior interacting with the aqueous cellular or extracellular environment, with the hydrophobic tail in the interior of the membrane. When a phospholipid is oxidised, a terminal non-polar methyl group is replaced with a polar carboxylate moiety (Figure 2C) [21]. Thus, the usually hydrophobic tail becomes hydrophilic, increasing the interaction with the aqueous cellular or extracellular environments, compromising membrane integrity and functions [21] and potentially leading to cell lysis at sufficiently high concentrations.

We also looked at the abundance of Oxo-PLs in response to mitochondrial and ER stress, both in wild-type and *MAPT* mutant cells. Both mitochondrial stress and ER stress could be driving Oxo-PL production, as evidenced by higher levels of these species in MAPT relative to wild-type neurons and in response to rotenone-induced mitochondrial stress (Figure 2B), although we cannot exclude other factors driving this response. However, whilst ER stress increases Oxo-PL levels in wild-type neurons (Figure 2B), we see a reduction in these species in response to tunicamycin treatment in *MAPT* mutants. Tunicamycin is a nucleoside antibiotic used to induce stress of the ER, via the unfolded protein response (UPR) [22]. The UPR is a cell survival mechanism which is initiated by an accumulation of misfolded or unfolded proteins in the ER, and serves to reduce the rate of protein translation, as well as degrading proteins already present [23]. However, chronic overactivation of the UPR can induce apoptosis, which is implicated in the pathology of neurodegeneration [24].

Tunicamycin-induced ER stress and acute UPR activation are detrimental to the health of dopamine neurons; however, the upregulation of this process could be helping to specifically reduce oxidative stress, and thus the abundance of Oxo-PLs. A possible mechanism through which this could be occurring is via the X-box binding protein 1 (XBP1), a major endoplasmic reticulum stress-linked transcriptional factor involved in the UPR. Though relatively understudied, research has evidenced XBP1 to be a regulator of oxidative stress [25]. Sado et al. investigated the IRE1α-XBP1 pathway in a mouse model, demonstrating that the exogenous expression of XBP1 protected dopamine neurons from cell death induced by a range of molecules used to mimic PD pathology and suggested that enhancement of XBP1 represents a potential therapeutic strategy [26]. Whilst the mechanisms by which XBP1 confers these protective effects is not fully understood, its role in regulating oxidative stress appear to be playing an important role. Knocking out the XBP1 gene has been shown to decrease the expression of several antioxidant genes including SOD1 and TRX1 [25]. SOD1 codes the protein superoxide dismutase, dysregulation of which has been linked to PD pathology [27], and is crucial to most forms of eukaryotic life as it converts superoxide (O_2_−) radicals into oxygen (O_2_) and hydrogen peroxide (H_2_O_2_). This is particularly important in the context of Oxo-PLs, as in aqueous solution superoxide exists in equilibria with its protonated hydroperoxyl (HO_2_−) form which plays a major role in lipid peroxidation [12].

In our data we saw lower levels of OXO-PLs in MAPT-N279K mutant dopamine neurons relative to wild-type controls which is contrary to what we might have expected to see (but not in direct contradiction to previous reports) given higher levels of oxidative stress in these cells. At this moment, we do not have a concrete explanation for these results, but we postulate that an unknown mechanism involving IRE1 activation by Oxo-PLs [28] increased the expression of XBP1, and in turn an increase in SOD1 expression and superoxide dismutase might explain these results.

### Study Limitations

Despite exhaustive efforts to remove confounding factors and ensure an optimal experimental design, the study still possess some limitations. Beyond the inherent limitations of working with iPSC models there are four key limitations:

Clonal variability: Genetic diversity amongst individual clones from a single parent line could lead to clonal specific results which suggests that clonal variability could be reflected. To minimize this possibility, we analysed two independent mutant lines and two independent isogenic control lines (in accordance with current best practices in the field of iPSC-modelling) and focused on conserved shifts in oxidized phospholipid abundance across lines. Whilst this reduces the likelihood of interpreting clonal-specific findings, it does not guarantee that our findings are not confounded by clonal variability with future studies required to validate the findings present in this study. Representation of mature dopamine neurons: In our previous work, we have demonstrated the dopamine neurons generated with this protocol match mature post-mortem dopamine neurons at the transcriptomic level. However, these in vitro neurons are unlikely to capture all the complex physiological features of their in vivo counterparts and stress response signatures in full. As a result, some of the phenotypes presented in this study might not recapitulate in full in the human brain.

Cellular localization of processes: In this study, we looked at mitochondrial and endoplasmic reticulum stress and ideally we would have also examined the cellular localization of processes to see if, for example, under ER stress, oxidation of phospholipids was happening within the ER or other cellular compartment. However, the lipids reported in this manuscript are most abundant in phospholipid bilayers and thus from post hoc analysis of this data we cannot make any specific inferences about the sub-cellular localization of effects.

Specificity of rotenone and tunicamycin: Rotenone and tunicamycin are well-validated methods for inducing mitochondrial and endoplasmic reticulum stress, respectively, in model systems and have been published in numerous studies. However, despite this, these compounds can also induce other cellular responses, meaning that we cannot be certain that the effects that we are observing are the result of mitochondrial and endoplasmic reticulum stress.

## 5. Conclusions

This study contributes to the understanding of metabolic abnormalities in tauopathy, by demonstrating that the *MAPT*-N279K mutation increases the abundance of oxidised phospholipids, with tunicamycin-induced ER stress appearing to reverse this effect in *MAPT* mutant neurons. Whilst speculative, the underlying mechanism in this effect may be associated with XBP1, which has been shown to have neuroprotective effects in models of PD and is an important regulator of oxidative stress via activation of SOD1 leading to increased clearance of superoxide radicals.

## Data Availability

All processed data will be available on request from the corresponding author. Data are not publicly available due to privacy or ethical restrictions.

## Supplementary Materials

The following supporting information can be downloaded at: https://www.mdpi.com/article/10.3390/antiox13050508/s1, Figure S1: PCA scores plot from initial analysis showing one sample accounting for 97.8% of total variance within the dataset (point was subsequently removed as an outlier).

## References

[1] Barbier P., Zejneli O., Martinho M., Lasorsa A., Belle V., Smet-Nocca C., Tsvetkov P.O., Devred F., Landrieu I. (2019). Role of Tau as a Microtubule-Associated Protein: Structural and Functional Aspects. Front. Aging Neurosci..

[2] Gong C., Iqbal K. (2008). Hyperphosphorylation of microtubule-associated protein tau: A promising therapeutic target for Alzheimer disease. Curr. Med. Chem..

[3] Pan L., Meng L., He M., Zhang Z. (2021). Tau in the Pathophysiology of Parkinson’s Disease. J. Mol. Neurosci..

[4] Fernandes H.J.R., Hartfield E.M., Christian H.C., Emmanouliou E., Zheng Y., Booth H., Bogetofte H., Lang C., Ryan B.J., Sardi S.P. (2016). ER stress and autophagic perterbations lead to elevated extracellular α-synuclein in GBA-N370S Parkinson’s iPSC-derived dopamine neurons. Stem Cell Rep..

[5] Zambon F., Cherubini M., Fernandes H.J.R., Lang C., Ryan B.J., Volpato V., Bengoa-Vergniory N., Vingill S., Attar M., Booth H.D.E. (2019). Cellular α-synuclein pathology is associated with bioenergetic dysfunction in Parkinson’s iPSC-derived dopamine neurons. Hum. Mol. Genet..

[6] Chen X., Guo C., Kong J. (2012). Oxidative stress in neurodegenerative diseases. Neural Regen. Res..

[7] Solleiro-Villavicencio H., Rivas-Arancibia S. (2018). Effect of Chronic Oxidative Stress on Neuroinflammatory Response Mediated by CD4(+)T Cells in Neurodegenerative Diseases. Front. Cell. Neurosci..

[8] Esteras N., Rohrer J.D., Hardy J., Wray S., Abramov A.Y. (2017). Mitochondrial hyperpolarization in iPSC-derived neurons from patients of FTDP-17 with 10+16 *MAPT* mutation leads to oxidative stress and neurodegeneration. Redox Biol..

[9] Bartolome F., Carro E., Alquezar C. (2022). Oxidative Stress in Tauopathies: From Cause to Therapy. Antioxidants.

[10] Teleanu D.M., Niculescu A., Lungu I.I., Radu C.I., Vladâcenco O., Roza E., Costăchescu B., Grumezescu A.M., Teleanu R.I. (2022). An Overview of Oxidative Stress, Neuroinflammation, and Neurodegenerative Diseases. Int. J. Mol. Sci..

[11] Sharifi-Rad M., Anil Kumar N.V., Zucca P., Varoni E.M., Dini L., Panzarini E., Rajkovic J., Tsouh Fokou P.V., Azzini E., Peluso I. (2020). Lifestyle, Oxidative Stress, and Antioxidants: Back and Forth in the Pathophysiology of Chronic Diseases. Front. Physiol..

[12] Ayala A., Muñoz M.F., Argüelles S. (2014). Lipid Peroxidation: Production, Metabolism, and Signaling Mechanisms of Malondialdehyde and 4-Hydroxy-2-Nonenal. Oxidative Med. Cell. Longev..

[13] Pizzino G., Irrera N., Cucinotta M., Pallio G., Mannino F., Arcoraci V., Squadrito F., Altavilla D., Bitto A. (2017). Oxidative Stress: Harms and Benefits for Human Health. Oxidative Med. Cell. Longev..

[14] Guo C., Sun L., Chen X., Zhang D. (2013). Oxidative stress, mitochondrial damage and neurodegenerative diseases. Neural Regen. Res..

[15] Fernandes H.J.R., Kent J.P., Bruntraeger M., Bassett A.R., Koulman A., Metzakopian E., Snowden S.G. (2023). Mitochondrial and Endoplasmic Reticulum Stress Trigger Triglyceride Accumulation in Models of Parkinson’s Disease Independent of Mutations in *MAPT*. Metabolites.

[16] Siddiqi F.H., Menzies F.M., Lopez A., Stamatakou E., Karabi C., Ureshino R., Ricketts T., Jimenez-Sanchez M., Estaban M.A., Lai L. (2019). Felodopine induces autophagy in mouse brains with pharmacokinetics amenable to repurposing. Nat. Commun..

[17] Kriks S., Shim J.W., Piao J., Ganat Y.M., Wakeman D.R., Xie Z., Carrillo-Reid L., Auyeung G., Antonacci C., Buch A. (2011). Dopamine Neurons derived from human ES cells efficiently engraft in animal models of Parkinson’s disease. Nature.

[18] Ebshiana A.A., Snowden S.G., Thambisetty M., Parsons R., Hye A., Legido-Quigley C. (2015). Metabolomic method: UPLC-q-ToF polar and non-polar metabolites in the healthy rat cerebellum using an in-vial dual extraction. PLoS ONE.

[19] (2022). R Core Team. https://www.R-project.org/.

[20] Bochkov V.N., Oskolkova O.V., Birukov K.G., Levonen A., Binder C.J., Stöckl J. (2010). Generation and biological activities of oxidized phospholipids. Antioxid. Redox Signal..

[21] Fruhwirth G.O., Loidl A., Hermetter A. (2007). Oxidized phospholipids: From molecular properties to disease. Biochim. Biophys. Acta (BBA)-Mol. Basis Dis..

[22] Wang Y., Zhang L., He Z., Deng J., Zhang Z., Liu L., Ye W., Liu S. (2020). Tunicamycin induces ER stress and inhibits tumorigenesis of head and neck cancer cells by inhibiting N-glycosylation. Am. J. Transl. Res..

[23] Hetz C. (2012). The unfolded protein response: Controlling cell fate decisions under ER stress and beyond. Nat. Rev. Mol. Cell Biol..

[24] Lam M., Marsters S.A., Ashkenazi A., Walter P. (2020). Misfolded proteins bind and activate death receptor 5 to trigger apoptosis during unresolved endoplasmic reticulum stress. eLife.

[25] Liu Y., Adachi M., Zhao S., Hareyama M., Koong A.C., Luo D., Rando T.A., Imai K., Shinomura Y. (2009). Preventing oxidative stress: A new role for XBP1. Cell Death Differ..

[26] Sado M., Yamasaki Y., Iwanaga T., Onaka Y., Ibuki T., Nishihara S., Mizuguchi H., Momota H., Kishibuchi R., Hashimoto T. (2009). Protective effect against Parkinson’s disease-related insults through the activation of XBP1. Brain Res..

[27] Trist B.G., Davies K.M., Cottam V., Genoud S., Ortega R., Roudeau S., Carmona A., De Silva K., Wasinger V., Lewis S.J.G. (2017). Amyotrophic lateral sclerosis-like superoxide dismutase 1 proteinopathy is associated with neuronal loss in Parkinson’s disease brain. Acta Neuropathol..

[28] Haberzettl P., Hill B.G. (2013). Oxidized lipids activate autophagy in a JNK-dependent manner by stimulating the endoplasmic reticulum stress response. Redox Biol..

