# Supplementary material for: Changes in Oxidised Phospholipids in Response to Oxidative Stress in Microtubule-Associated Protein Tau (MAPT) Mutant Dopamine Neurons
Source: Antioxidants (Basel). 2024 Apr 24;13(5):508. doi: 10.3390/antiox13050508 (PMC11118013; doi:10.3390/antiox13050508)
Supplement: Supplementary file 1 [file antioxidants-13-00508-s001.zip › antioxidants-2948244-supplementary.pdf]

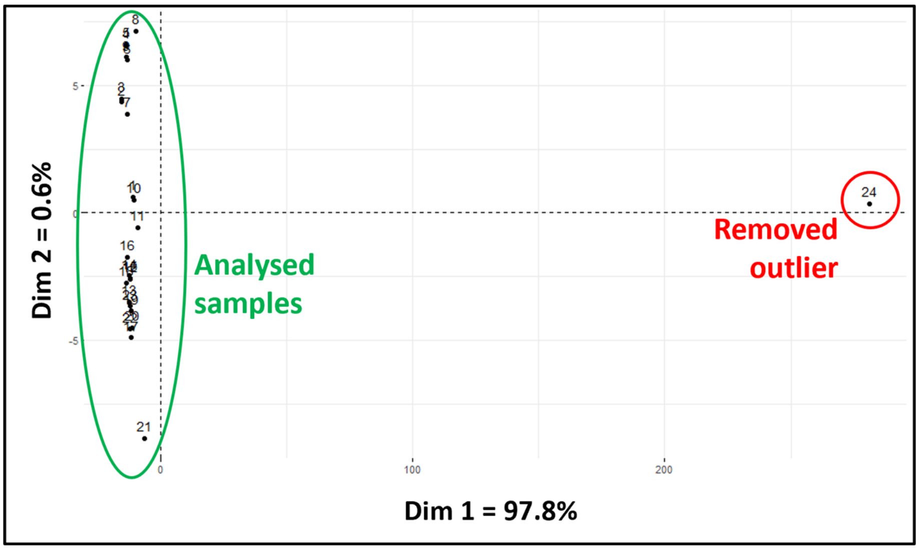

Supplemetanl Figure 1 PCA scores plot from initial analysis showing one sample accounting for 97. 8% of total variance within the dataset (point was subsequently removed as an outlier).
